# Osseous Growths in the Gums

**Published:** 1869-06

**Authors:** Thruston Wolfe


### ARTICLE III.

### *Osseous Growths in the Gums.*

By Dr. Thruston Wolfe.

The first case which came under my observation was that of a colored girl, fifteen years of age, who applied to me for the purpose of having (what she believed to be) a tooth extracted.

On examining her mouth I found all of her teeth perfectly sound and the gums in a healthy condition. But between the inferior right second bicuspid and the first molar, I discovered an osseous growth on the lingual surface of the gum below the necks of the teeth. By means of an elevator I dislodged it without difficulty, as it appeared to be connected with a cartilaginous substance, and not with the bone. Very little hæmorrhage followed its removal, and the pain of the operation was but slight.

In size this osseous growth was nearly half an inch long and a quarter of an inch wide. A careful examination (without a microscope however) convinced me that it was a true osseous growth.

The second case I met with was that of a little girl between nine and ten years old. The tumor in this case was situated between the superior right second bicuspid and the first molar, on the palatine surface of the gum, having an irregular shape and extending down between the teeth named as far as their buccal surfaces.

I removed this growth in the same manner as the first, and with the same results.

The third case was that of a lady aged about forty-five years, who complained that something in her mouth had been annoying her for years. On examining her mouth I found precisely the same kind of a growth as in the second case I have mentioned, except that it was situated between the superior second and third molars, far up on the palatine surface of the gum.

I removed this growth also, and it appeared to have only a fleshy connection. Very little pain and but slight hæmorrhage followed its removal.

In all of the cases I have described the gums presented a perfectly healthy appearance, and the teeth were free from salivary calculus. Before removing the osseous growth in the second case I have mentioned, I called in a physician to examine it, and he pronounced it to be a protrusion of the alveolar process, but remarked that he had never seen anything like it before.

They were all certainly true osseous growths and neither teeth nor roots of teeth, as I examined them very carefully to discover whether they were of the class known as "supernumerary."

*Remarks.*—The three cases above described are of considerable interest, as Dr. Wolfe is satisfied from careful examinations that these growths were truly osseous in their nature, and neither supernumerary teeth nor roots of teeth imbedded in the gums.

What are known as "dentigerous cysts" are occasionally met with, and may occur in either jaw, but as these are always connected with the roots of fully developed teeth or with imperfectly developed teeth, and arise in connection with teeth which have from some cause remained within the jaw, or inverted teeth giving rise to more or less irritation, the cases under consideration were clearly not of this character. Besides, dentigerous cysts are connected with the maxillary bones, being inserted between the two plates of the bone, and contain a serous glairy fluid, often invading the antrum, when in the upper jaw, by absorption of the bony wall.

We think, therefore, that these cases may be properly classed under the term "epulis", as the abnormal growths of the gums known by this name vary in their nature.

Ordinarily, epulis consists of a firm fibrous tumor in which we sometimes find fibro-plastic cells, and, occasionally, a development of true osseous matter. A case is mentioned by Christopher Heath, F. R. C. S., where he removed a large epulis from the upper jaw of a young woman, in which a nodule of bone of a large size was developed near the surface of the gum and quite unconnected with the alveolus.

The surfaces of these tumors are often covered by perfectly healthy mucous membrane which is stretched over them, and, although they may have no immediate connection with the teeth, are probably caused by some irritation proceeding from these organs. Their growth is also slow, and the patient is only aware of their existence by the deformity they occasion. [Ed.]

